# Ginsenoside Re Improves Inflammation and Fibrosis in Hepatic Tissue by Upregulating PPAR*γ* Expression and Inhibiting Oxidative Stress in db/db Mice

**DOI:** 10.1155/2021/9003603

**Published:** 2021-10-08

**Authors:** Yichuan Jiang, Dayun Sui, Min Li, Huali Xu, Xiaofeng Yu, Jinsha Liu, Qian Yu

**Affiliations:** ^1^Department of Pharmacy, China-Japan Union Hospital, Jilin University, Changchun 130033, China; ^2^Department of Pharmacology, School of Pharmaceutical Sciences, Jilin University, Changchun 130021, China; ^3^Pharmacological Experiment Center, School of Pharmaceutical Sciences, Jilin University, Changchun 130021, China; ^4^Department of Cardiology, China-Japan Union Hospital, Jilin University, Changchun 130033, China

## Abstract

Ginsenoside Re (Re) is the main component of “Zhenyuan Capsule” (ZYC), which was wildly used in clinic in China for adjunctive treatment of coronary heart disease (CHD) and type II diabetes (T2DM). Nonalcoholic fatty liver disease (NAFLD) is one of the most important complications of T2DM, as well as an important risk factor of CHD. The aim of the present study was to investigate the effects of Re on NAFLD in db/db mice, one of the most recognized gene deficient animal models on T2DM. Sixteen db/db mice and sixteen wild-type mice were divided into four groups and orally administered Re or placebo in equal volume. According to the results, Re showed no obvious effect on blood glucose, lipids, or body weight of db/db mice. Histology pictures of hepatic tissue showed that Re did not improve steatosis, too. However, some evidence suggested that hepatic injury in db/db mice was attenuated by Re administering. Collagen deposition and aminotransferase elevation were significantly downregulated in the DB + Re group compared to those in the DB Group. The mechanisms of the protect effects of Re represented in db/db mice with NAFLD might be inhibiting oxidative stress and the reupregulation of peroxisome proliferator-activated receptor *γ* (ppar*γ*) expression. The results of this study indicated that ZYC might be able to help T2DM patients with NAFLD to control the progress of NAFLD as an alternation of thiazolidinediones, synthetic agonists of PPAR*γ*, whose side effects and adverse events should not be ignored.

## 1. Introduction

**Figure 1:**
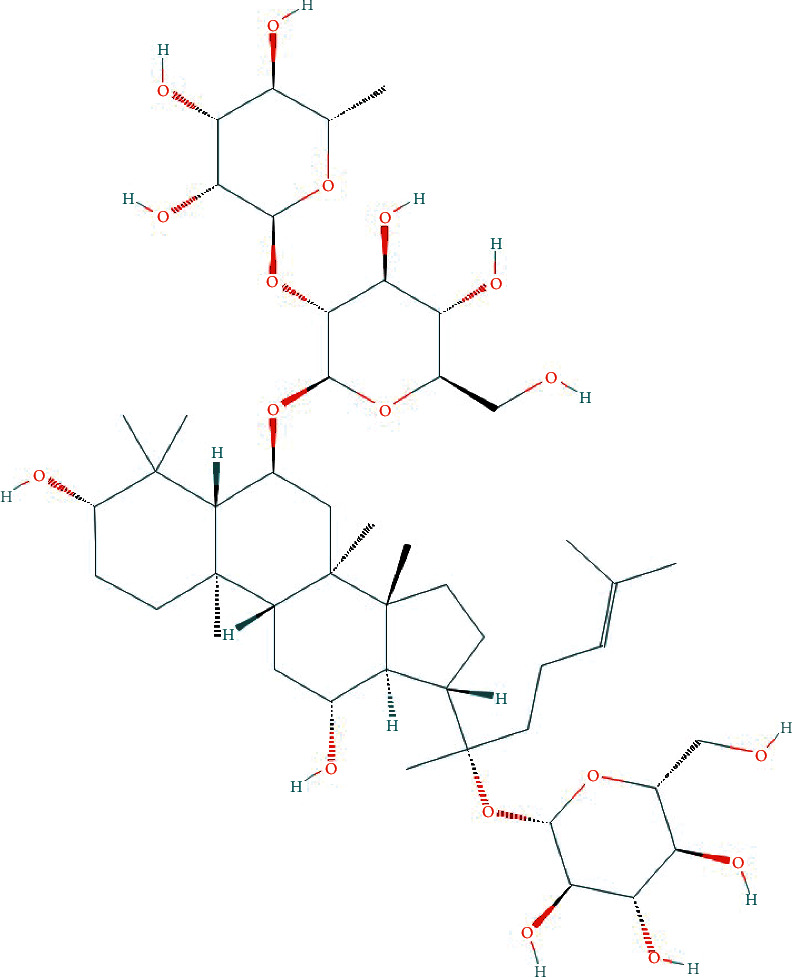
Chemical structure of Re, downloaded from PubChem (https://www.pubchem.ncbi.nlm.nih.gov), CID 441921.

Ginsenoside Re (Re) is one of the protopanaxatriol-type saponins (Figure 1), which are abundant in Araliaceae plants such as *Panax ginseng* C.A. Meyer (ginseng) and *Panax quinquefolium* L. (American ginseng). Especially, Re is the essential component of total saponins extracted from the fruit of ginseng, which had been developed as a drug named “Zhenyuan Capsule” (ZYC). ZYC was wildly used in clinic in China for adjunctive treatment of coronary heart disease (CHD) and type II diabetes (T2DM), especially CHD with abnormal glucose and lipid metabolism [1]. Current evidence showed that ZYC combined with routine treatment could improve the cardiac function and quality of life of patients with chronic heart failure, and with high safety [2]. The content of Re is also the major technical standard in the quality control of ZYC.

Our group is currently undertaking a long-term study assessing the effects of various ginsenosides, including Re, in various animal models of chronic disease, such as hypertension [3, 4], CHD [5–7], and T2DM. Especially, we had demonstrated that Re improves acute myocardial ischemia-mediated myocardial injury, myocardial fibrosis, and heart failure. Regarding the effects of Re on CHD, the mechanisms might be associated with inhibiting oxidative stress and transforming growth factor-*β*- (TGF-*β*-) mediated fibrosis in the myocardium in rats [6, 7]. As Re is the essential component of ZYC, which has been widely used as an adjunctive treatment of CHD and T2DM, in this study, we determined the protective effects of Re on nonalcoholic fatty liver disease (NAFLD), which has a complex relationship with CHD and T2DM.

Studies have shown that, up to 70% of patients with diabetes might present NAFLD, which often results in abnormal liver function [8–10]. Studies have also demonstrated that NAFLD is an important risk factor for higher prevalence of cardiovascular diseases, for example, CHD [9, 11]. According to the “multihit hypothesis” of NAFLD [12–16], insulin resistance within the liver is implicated in the pathogenesis of NAFLD. Upregulation of the peroxisome proliferator-activated receptor gamma (PPAR*γ*) pathway could attenuate inflammation and fibrosis in hepatic tissue as well as insulin resistance, and these had been confirmed by preclinic animal research in *vivo* or clinic research [8, 17, 18]. Thiazolidinediones (TZDs), synthetic agonists of PPAR*γ*, are widely used in clinic for improving hepatic injury in NAFLD patients [11, 19–21].

PPAR*γ* agonists have been demonstrated to improve hepatic inflammation and fibrosis in T2DM animal models with NAFLD [22, 23]. In this study, we were going to observe the hepatic protective effects of Re and determined the expression of PPAR*γ* in hepatic tissue in db/db mice (BKS-Leprem2Cd479/Gpt), one of the most recognized gene deficient animal models on T2DM with the complication of NAFLD. The leptin receptor is deficient in db/db mice, which results in obesity, hyperglycemia, hyperlipidemia, and hepatic steatosis [24, 25]. Wild-type (WT) mice with the same genetic background (C57BLKS/JGpt) were used in the two control groups. The effects of Re on oxidative stress, inflammation, and fibrosis in hepatic tissue were also determined, as we had demonstrated that Re improves myocardial injury in CHD by inhibiting oxidative stress, inflammation, and fibrosis [6, 7].

## 2. Materials and Methods

### 2.1. Chemicals and Reagents

Re (95% purity) was obtained from Dr. Yanping Chen at the Department of Natural Medicinal Chemistry, School of Chemistry, Jilin University. It was dissolved in 0.5% sodium carboxymethyl cellulose solution (0.5% CMC-Na) for use. All of the other chemicals and reagents were of analytical grade.

### 2.2. Animals

16 db/db (abbreviated as DB in group names) mice and 16 WT mice were purchased from Nanjing Biomedical Research Institute of Nanjing University (Nanjing, China). All of the mice were male and 12∼13-weeks old. The experimental animal house was of specific pathogen free (SPF) grade. The constant temperature in the house was 22–24°C, and the relative humidity was 45–55%. All the mice had free access to water and food. The light/dark cycle was 12 hours/12 hours.

### 2.3. Experimental Protocols

All the mice were divided into 4 groups: (I) group WT, 8 WT mice; (II) group WT + Re, 8 WT mice; (III) group DB, 8 db/db mice; and (IV) group DB + Re, 8 db/db mice. Mice in the II and IV groups were administered Re at the doze of 30 mg kg^−1^ d^−1^ orally, while mice in the I and III groups were administered 0.5% CMC-Na as placebo orally. In our previous studies [6, 7], rats were orally administered 20* *mg kg^−1^ d^−1^ Re. Therefore, the biological equivalent dose of Re in mice is 30 mg kg^−1^ d^−1^. Administration of placebo or Re was carried on for 8 weeks. During these 8 weeks, blood glucose and body weight of mice were measured weekly.

After the 8-week treatment, all the mice in the 4 groups were sacrificed following the blood sample collection. Then, the tissue samples of the livers were obtained. The blood samples of mice were prepared into serum by centrifugation (1000 g, 4°C for 15 minutes) and kept at −80°C. The hepatic tissue samples were fixed in 4% formaldehyde or snap-frozen with liquid nitrogen. The samples fixed in formaldehyde were embedded in paraffin, and the snap-frozen samples were kept at −80°C.

### 2.4. Blood Glucose Measurement

Lateral tail vein blood glucose was measured using the Fasting Blood Glucose Test Kit (GlucoLab, Infopia Co., Ltd., Kyunggi, Korea) following the manufacturer's protocol weekly during the 8-week treatment.

### 2.5. Serum Biochemical Assays

Biochemical assay kits were purchased form Nanjing Jiancheng Bioengineering Institute (Nanjing, China): ALT (alanine aminotransferase, C009-2-1), AST (aspartate aminotransferase, C010-2-1), TG (triglyceride, A110-1-1), TC (total cholesterol, A111-1-1), HDL (high-density lipoprotein cholesterol, A112-1-1), and LDL (low-density lipoprotein cholesterol, A113-1-1). The activities of ALT and AST and levels of TG, TC, HDL, and LDL in serum were assayed following the manufacturer's protocols.

### 2.6. Hepatic Tissue Biochemical Assays

Hepatic tissue samples kept at −80°C were homogenized with ice-cold normal saline (100 mg: 900 *μ*L), and the supernatant samples were prepared for biochemical assays by centrifuging (1000 g, 4°C for 15 minutes). Biochemical assay kits were purchased form Nanjing Jiancheng Bioengineering Institute (Nanjing, China): MDA (malondialdehyde, A003-1-1), SOD (superoxide dismutase, A001-3-1), CAT (catalase, A007-1-1), and GSH-Px (glutathione peroxidase, A005-1-2). The levels of MDA and activities of SOD, CAT, and GSH-Px in hepatic tissue were assayed following the manufacturer's protocols.

### 2.7. Histopathological Assessment

Hepatic tissue samples embedded in paraffin were cut into 4*μ*m-thick sections for hematoxylin and eosin (H&E) or Masson trichrome (Masson) staining. The Masson kit (BSBA-4079A, ZSGB-BIO, Beijing, China) was used following the manufacturer's protocols for Masson staining.

Hepatic tissue samples kept at −80°C were cut into 6*μ*m-thick cryostat sections for Oil-Red-O (ORO) staining of lipid droplets. The ORO kit (BSBA-4081, ZSGB-BIO, Beijing, China) was used following the manufacturer's protocols for ORO staining.

All the stained sections were examined using a Nikon E100 light microscope (Nikon Corporation, Tokyo, Japan); then, histopathology pictures were captured.

### 2.8. Immunohistochemistry

Staining of immunohistochemistry (IHC) was performed using the two-step rabbit IHC kit (PV-9001, ZSGB-BIO, Beijing, China) and DAB kit (ZLI-9017, ZSGB-BIO, Beijing, China) following the manufacturer's protocols. Primary antibodies used in staining were anti-PPAR-*γ* (bs-4590R, Bioss Inc., Beijing, China) and anti-TGF-*β*1 (bs-0103R, Bioss Inc., Beijing, China). All the stained sections were examined using the microscope mentioned above; then, histopathology pictures were captured and further analyzed using Image Pro Plus 6.0 (Media Cybernetics, Inc., Rockville, MD, USA).

### 2.9. RNA Purification and RT-qPCR

Total RNA was isolated from frozen hepatic tissue samples using TRIzol reagent (Thermo Fisher Scientific, Inc., MA, USA) following the manufacturer's protocol. Total RNA was reverse-transcribed, and qPCR was conducted using the TransScript Green Two-Step qRT-PCR SuperMix (TransGen Biotech Co., Ltd., Beijing, China) on a Stratagene Mx3000P Real-Time PCR System (Agilent Technologies, Inc., CA, USA). The relative fold changes in the mRNA levels of the target genes, including tumor necrosis factor-*α* (TNF-*α*), interleukin-6 (IL-6), procollagen I (Col-I), and procollagen III (Col-III), were determined using the 2^−ΔΔCt^ method [26]. *β*-Actin was used as a housekeeping gene. All primers are listed in Table 1.

### 2.10. Statistical Analysis

SPSS 16.0 (IBM Corporation, NY, USA) was employed for all statistical analyses. Data are mean ± standard deviation (SD). One-way analysis of variance (ANOVA) with Tukey's post hoc test was employed for group comparisons, with *P* < 0.05 indicating statistical significance.

## 3. Results

### 3.1. Re Has No Significant Effect on Body Weight, Blood Glucose, and Lipids

**Figure 2:**
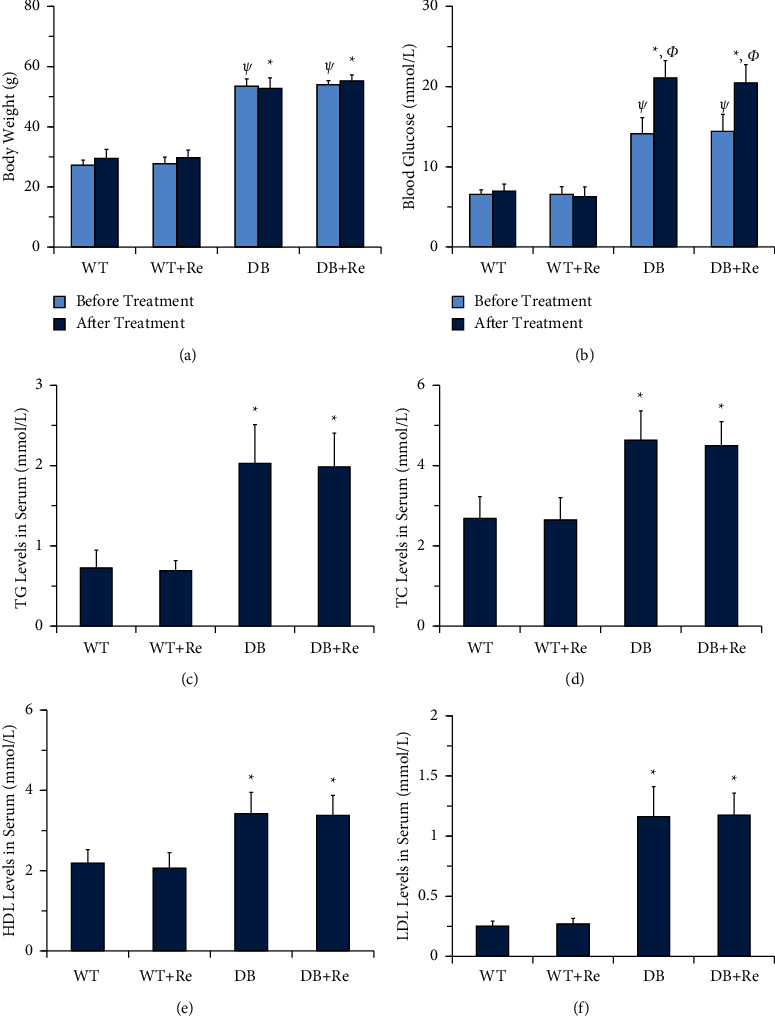
Re has no significant effect on body weight, blood glucose, and lipids. (a) Body weight of mice prior to and following eight weeks of treatment; (b) blood glucose of mice prior to and following eight weeks of treatment; (c) TG levels in serum in mice after treatment; (d) TC levels in serum in mice after treatment; (e) HDL levels in serum in mice after treatment; and (f) LDL levels in serum in mice after treatment. Data are mean ± SD, and *n* = 8. One-way ANOVA with Tukey's post hoc test was employed for group comparisons, with *P* < 0.05 indicating statistical significance. Ψ, *P* < 0.05 compared to the WT group before treatment; ^*∗*^*P* < 0.05 compared to the WT group after treatment; Φ, *P* < 0.05 compared to the DB group before treatment; and Ω, *P* < 0.05 compared to the DB + Re group before treatment.

The mice in the WT group and WT + Re group did not present a significant difference regarding body weight, blood glucose, and four kinds of lipids (TG, TC, HDL, and LDL). The situation was the same between the DB group and DB + Re group. But, the levels of body weight, blood glucose, and lipids are all significantly higher in db/db mice than in WT mice, no matter being administered of Re or not (Figure 2).

### 3.2. Re Reduces Activities of Aminotransferases in Serum

**Figure 3:**
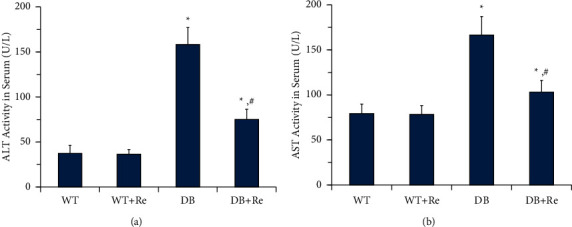
Re reduces activities of aminotransferases in serum. (a) Activities of ALT in serum; (b) activities of AST in serum. Data are mean ± SD, and *n* = 8. One-way ANOVA with Tukey's post hoc test was employed for group comparisons, with *P* < 0.05 indicating statistical significance. ^*∗*^*P* < 0.05 compared to the WT group; ^#^*P* < 0.05 compared to the DB group.

In the DB group, activities of ALT and AST in serum were significantly higher than the activities in the WT Group. The disorder of glucose and lipid metabolism resulted in hepatocytes injury and aminotransferase leakage in db/db mice. Comparing to the DB group, Re administration markedly reduced the activities in the DB + Re group, while the activities in the WT + Re group were not significantly downregulated comparing to those in the WT group (Figure 3). These results indicated that Re administration attenuated hepatocytes injury and aminotransferase leakage in db/db mice.

### 3.3. Re Inhibits Oxidative Stress in Hepatic Tissue

**Figure 4:**
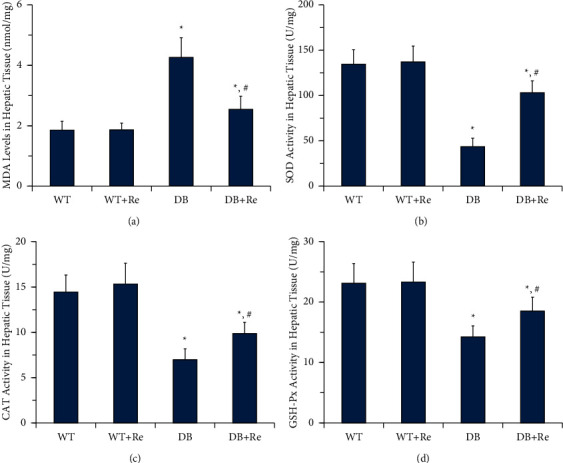
Re inhibits oxidative stress in hepatic tissue. (a) MDA levels in hepatic tissue; (b) activities of SOD in hepatic tissue; (c) activities of CAT in hepatic tissue; and (d) activities of GSH-Px in hepatic tissue. Data are mean ± SD, and *n* = 8. One-way ANOVA with Tukey's post hoc test was employed for group comparisons, with *P* < 0.05 indicating statistical significance. ^*∗*^*P* < 0.05 compared to the WT group; ^#^*P* < 0.05 compared to the DB group.

Compared to the WT group, the activities of SOD, CAT, and GSH-Px in hepatic tissue in the DB group were significantly downregulated, while the MDA levels in the DB group were significantly upregulated. These changes indicated that oxidative stress existed in hepatic tissue of db/db mice. Oxidative stress was inhibited by Re administration, as the activities of SOD, CAT, and GSH-Px were significantly upregulated and MDA levels were significantly downregulated in the DB + Re group, compared to those in the DB Group. There was no significant difference in the results of these biochemical tests between the WT group and WT + Re group (Figure 4).

### 3.4. Re Does Not Reverse Simple Steatosis but Inhibits Fibrosis in Hepatic Tissue

**Figure 5:**
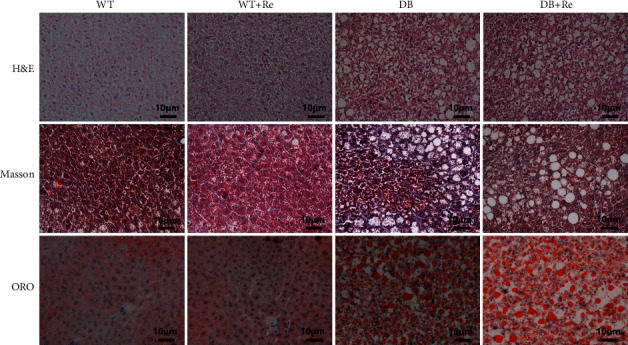
Re does not reverse simple steatosis but inhibits fibrosis in hepatic tissue. Representative H&E, Masson, and ORO staining histology photomicrographs of the hepatic tissue in mice. All scale bars represent 10 *μ*m.

According to the results of H&E, Masson, and ORO staining examination, the hepatic tissue specimens in both the DB group and DB + Re group represented obvious steatosis. The morphology of hepatic lobules was also disordered in both of these two groups. All of these pathological changes did not exist in the WT group or WT + Re group. However, the Masson staining examination showed that collagen was deposited in hepatic tissue in the DB group, while it was markedly improved in the DB + Re group (Figure 5). These results demonstrated that Re has an antifibrosis effect on the liver of db/db mice, but Re does not reverse simple steatosis.

### 3.5. Re Upregulates PPAR*γ* and Downregulates TGF-*β*1 Expression in Hepatic Tissue

According to the IHC examination, the expression levels of PPAR*γ* were significantly higher in hepatic tissue specimens from the DB group than those from the WT Group. They were compensated upregulated because of glucose and lipid metabolism. Interestingly, the levels were even further upregulated in the DB + Re group by Re administration, improving insulin resistance, inflammation, and fibrosis.

The situation of TGF-*β*1 was different. As an important factor on inflammation and fibrosis, TGF-*β*1 was over expressed in hepatic tissue in mice from the DB group, compared to that from the WT group. Also, the expression of TGF-*β*1 was significantly downregulated by Re administration in the DB + Re group, compared to that in the DB group.

**Figure 6:**
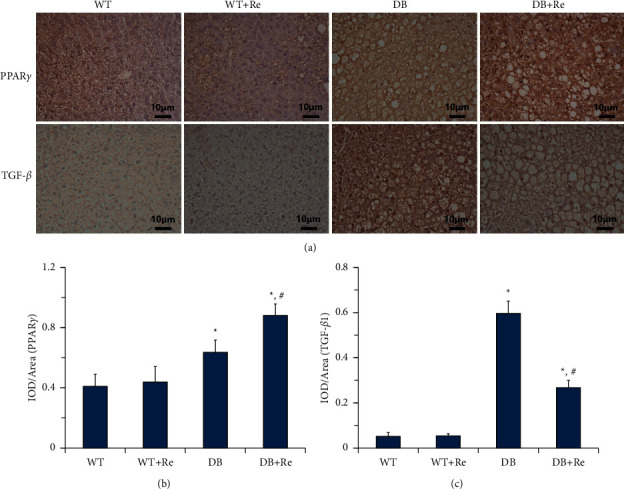
Re upregulates PPAR*γ* and downregulates TGF-*β*1 expression in hepatic tissue. (a) Representative IHC staining photomicrographs of the hepatic tissue in mice and quantitative results of IHC staining, which were presented as IOD/area and were proportional to the levels of PPAR*γ* (b) and TGF-*β*1 (c). All scale bars represent 10 *μ*m. Data are mean ± SD, and *n* = 4. One-way ANOVA with Tukey's post hoc test was employed for group comparisons, with *P* < 0.05 indicating statistical significance. ^*∗*^*P* < 0.05 compared to the WT group; ^#^*P* < 0.05 compared to the DB group.

The expression levels of PPAR*γ* or TGF-*β*1 in hepatic tissue specimens from the WT + Re group did not represent a significant difference compared to those from the WT group (Figure 6).

### 3.6. Re Reduces Levels of Inflammatory Cytokines and Procollagens in Hepatic Tissue

**Figure 7:**
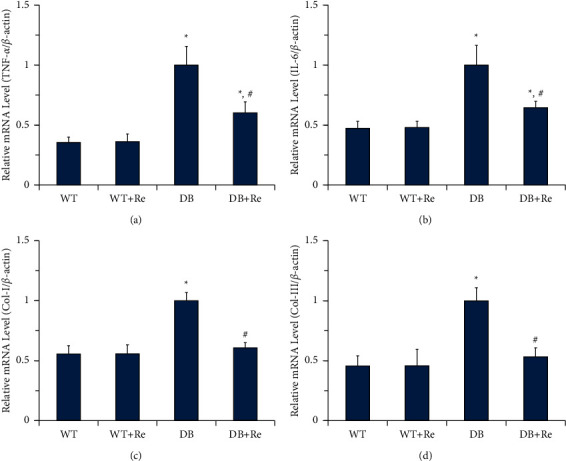
Re reduces levels of inflammatory cytokines and procollagens in hepatic tissue. Relative mRNA levels of TNF-*α* (a), IL-6 (b), Col-I (c), and Col-III (d). *β*-Actin was used as a housekeeping gene. Data are mean ± SD, and *n* = 4. One-way ANOVA with Tukey's post hoc test was employed for group comparisons, with *P* < 0.05 indicating statistical significance. ^*∗*^*P* < 0.05 compared to the WT group; ^#^*P* < 0.05 compared to the DB group.

RT-qPCR was used to measure the relative levels of mRNA in hepatic tissue (Figure 7), including two inflammatory cytokines (TNF-*α* and IL-6) and two procollagens (Col-I and Col-III). The levels of TNF-*α*, IL-6, Col-I, and Col-III were all significantly higher in the DB group, compared to those in the WT group, while those levels from the WT + Re group did not represent a significant difference compared to those from the WT group. The levels of two inflammatory cytokines were significantly downregulated in the DB + Re group compared to those in the DB group, but were still significantly higher compared to those in the WT Group. The levels of two procollagens were also significantly downregulated by Re administration in the DB + Re group, and they did not represent significant difference compared to those in the WT Group.

## 4. Discussion

As it is mentioned above, NAFLD is one of the most common causes of abnormal liver function among adults. It is also one of the most common and important complications of T2DM, as well as an important risk factor of CHD. NAFLD usually develops from simple steatosis to nonalcoholic steatohepatitis (NASH), which can progress to end-stage liver disease [9]. Thus, it is important to improve early hepatic injury and prevent simple steatosis from developing into NASH or hepatic fibrosis.

ZYC, whose essential component is Re, has been widely used for T2DM and CHD treatment in clinic in China. Though NAFLD/NASH has a complex relationship with CHD and T2DM, few studies have been reported regarding using ZYC for NAFLD/NASH treatment. So, this study was performed to confirm that whether Re, an essential component of ZYC, could improve NAFLD in db/db mice.

As the results in this study showed, although Re did not affect the body weight, blood glucose, and lipids of db/db mice (Figure 2), it did control the progress of NAFLD, as the activities of aminotransferases in serum were reduced (Figure 3) and collagen deposition in hepatic tissue was attenuated (Figure 5).

As the first stage of NAFLD, simple steatosis in liver is inevitable in most T2DM patients, which also exist in db/db mice. According to the “multihit hypothesis” of NAFLD [12–16], insulin resistance within the liver is implicated in the pathogenesis of NAFLD. TZDs, synthetic agonists of PPAR*γ*, are the only current antidiabetic agents that increase insulin sensitivity as the main mechanism. Although TZDs have recently fallen into disuse in glycemic control due to concerns over side effects and adverse events, it is worthy to use them in T2DM patients with NAFLD [19, 27, 28]. In this study, Re upregulated the expression of PPAR*γ* in hepatic tissue in db/db mice (Figures 6(a) and 6(b)), and this might contribute to the effects of Re which improves inflammation and fibrosis in hepatic tissue (Figures 5 and 7). These effects were not able to reverse simple steatosis in hepatic tissue in db/db mice, but did prevent it from developing into NASH or hepatic fibrosis (Figures 3 and 5).

In our previous studies [6, 7], we had demonstrated that Re improved CHD by reducing oxidative stress and TGF-*β* levels, which are both important factors of the progress of NAFLD [14, 28, 29]. In this study, we demonstrated that Re reduced oxidative stress (Figure 4) and TGF-*β*1 levels (Figures 6(a) and 6(c)), and these might also contribute to the anti-inflammation, antifibrosis, and hepatic protective effects of Re.

According to global research for decades, it is well recognized that ginsenosides, such as Re, improve a variety of cardiovascular and metabolic diseases, as well as their complication. The most common mechanisms were the antioxidation, anti-inflammation, and antifibrosis effects of ginsenosides, while the activation of PPAR*γ* was also reported [30]. These were consistent with our findings in this study.

## 5. Conclusions

Although no obvious effect on simple steatosis in the livers of db/db mice was observed, Re indeed attenuates early hepatic injury, including mild ALT/AST elevation and collagen deposition. The mechanisms of improving inflammation and fibrosis in hepatic tissue might be associated to the upregulation of PPAR*γ* expression and downregulation of oxidative stress. These indicated that ZYC, whose essential component is Re, might be able to help T2DM patients with NAFLD to control the progress of NAFLD as an alternation of TZDs, whose side effects and adverse events should not be ignored. Further studies and clinical trials are needed to be carried out before using in clinic.

## Figures and Tables

**Table 1:** Primer sequence for RT-qPCR.

Gene		Sequence (5'-3')
*β*-Actin	Forward	GGCTGTATTCCCCTCCATCG
	Reverse	CCAGTTGGTAACAATGCCATGT

TNF-*α*	Forward	GTCGTAGCAAACCACCAAGT
	Reverse	TGTGGGTGAGGAGCACGTAG

IL-6	Forward	GTCCTTCAGAGAGATACAGAAACT
	Reverse	AGCTTATCTGTTAGGAGAGCATTG

Col-I	Forward	CTTCACCTACAGCACCCTTGTG
	Reverse	TGACTGTCTTGCCCCAAGTTC

Col-III	Forward	TGTCCTTTGCGATGACATAATCTG
	Reverse	AATGGGATCTCTGGGTTGGG

## Data Availability

The data used to support the findings of this study are included within the article.
